# Interaction of TGFβ and BMP Signaling Pathways during Chondrogenesis

**DOI:** 10.1371/journal.pone.0016421

**Published:** 2011-01-28

**Authors:** Bettina Keller, Tao Yang, Yuqing Chen, Elda Munivez, Terry Bertin, Bernhard Zabel, Brendan Lee

**Affiliations:** 1 Department of Molecular and Human Genetics, Baylor College of Medicine, Houston, Texas, United States of America; 2 Howard Hughes Medical Institute, Baylor College of Medicine, Houston, Texas, United States of America; 3 Center of Pediatrics and Adolescence Medicine, University Hospital of Freiburg, Freiburg, Germany; Brigham and Women's Hospital, United States of America

## Abstract

TGFβ and BMP signaling pathways exhibit antagonistic activities during the development of many tissues. Although the crosstalk between BMP and TGFβ signaling pathways is well established in bone development, the relationship between these two pathways is less well defined during cartilage development and postnatal homeostasis. We generated hypomorphic mouse models of cartilage-specific loss of BMP and TGFβ signaling to assess the interaction of these pathways in postnatal growth plate homeostasis. We further used the chondrogenic ATDC5 cell line to test effects of BMP and TGFβ signaling on each other's downstream targets. We found that conditional deletion of *Smad1* in chondrocytes resulted in a shortening of the growth plate. The addition of *Smad5* haploinsufficiency led to a more severe phenotype with shorter prehypertrophic and hypertrophic zones and decreased chondrocyte proliferation. The opposite growth plate phenotype was observed in a transgenic mouse model of decreased chondrocytic TGFβ signaling that was generated by expressing a dominant negative form of the TGFβ receptor I (ΔTβRI) in cartilage. Histological analysis demonstrated elongated growth plates with enhanced *Ihh* expression, as well as an increased proliferation rate with altered production of extracellular matrix components. In contrast, in chondrogenic ATDC5 cells, TGFβ was able to enhance BMP signaling, while BMP2 significantly reduces levels of TGF signaling. In summary, our data demonstrate that during endochondral ossification, BMP and TGFβ signaling can have antagonistic effects on chondrocyte proliferation and differentiation *in vivo*. We also found evidence of direct interaction between the two signaling pathways in a cell model of chondrogenesis *in vitro*.

## Introduction

The transforming growth factor β (TGFβ) superfamily is a large group of secreted polypeptides, including TGFβ1, 2, 3, activins, inhibins and nodal, bone morphogenetic proteins (BMPs) and growth differentiation factors (GDFs). TGFβ family members are vitally important during embryogenesis, as well as in the homeostasis in the adult tissues. These molecules are also involved in the regulation of cellular processes such as proliferation, differentiation and apoptosis.

The Smad-dependent TGFβ and BMP signaling pathways have been well established for decades. TGFβ or BMP ligands bind to specific type II receptors to recruit the corresponding type I receptor to initiate a cascade of events leading to phosphorylation of their specific receptor-Smads (R-Smads). Generally, TGFβ signaling depends on Smad2 and Smad3, while BMP signaling depends on Smad1, 5 and 8. The phosphorylated R-Smad forms a heterocomplex with the Smad4, the common partner Smad (Co-Smad). The R-Smad/Co-Smad complex then translocates into the nucleus, where it binds to promoters of target genes and regulates their transcription [1]. Besides, TGFβ/BMP signaling can also be mediated by noncanonical MAPK pathways, such as P38, JNK and Erk1/2 signaling pathways, during chondrogenesis [2].

BMPs play an important role in the earliest stages of chondrogenesis, i.e. mesenchyme condensation and cell fate determination. *In vitro* BMPs can promote mesenchymal cells to differentiate into chondrocytes in high-density cultures [3] in part by inducing *Sox9* gene expression [4]. Not surprisingly, BMP signaling components are highly expressed in growth plates with specific temporal-spatial patterns that correlate with functions during growth plate development and homeostasis. [5], [6], [7], [8], [9], [10]. For example, BMPs play an important role in regulating the proliferation of chondrocytes. Loss of Noggin, a potent BMP antagonist, leads to overgrowth of skeletal elements in mice [11]. On the other hand, misexpression of Noggin in chick limbs causes reduction in of skeletal elements [6]. Moreover, BMPs promote the differentiation of proliferating chondrocytes to hypertrophic chondrocytes, the chondrocyte specific expression of constitutively active *Bmpr-1a* in transgenic mice accelerated the maturation and hypertrophy of proliferating chondrocytes [12]. Recently, Retting et al. showed that the complete loss of Smad1 and Smad5 in chondrocytes leads to a severe chondrodysplasia and that both mediators have overlapping functions in the developing growth plate. However, only one copy of either Smad1 or Smad5 is sufficient to correct the chondrodysplasia seen in the chondrocyte specific Smad1^−/−^/Smad5^−/−^ growth plate [13].

The importance of TGFβ during skeletal development has been demonstrated in various models. TGFβ1, 2 and 3 are expressed in the mouse perichondrium and periosteum from E13.5 until after birth [14], [15], [16], [17], [18], while TGFβ receptors are expressed in perichondrium and in chondrocytes [19]. The growth plate of Smad3^−/−^ mice exhibits the formation of large osteophytes, decreased production of proteoglycans. Moreover, the Smad3^−/−^ mice develop degenerative joint disease resembling human osteoarthritis, featuring an increased expression of Collagen X in the chondrocytes of synovial joints [20]. Additionally, the conditional deletion of the *TgfbrII* with *Col2a1-Cre* and *Prx1-Cre* causes axial skeleton defects, alteration in hypertrophic differentiation in growth plates, and joint fusions in phalanges [21], [22], supporting a role of the TGFβ signaling pathway in both axial and appendicular bone and joint formation.

To further understand the role of BMP and TGFβ signaling in the postnatal growth plate and to elucidate potential crosstalk between both signaling pathways we generated and compared two mouse models with partial deficiency of either BMP or TGFβ signaling in the growth plate. Our findings showed that while reduced BMP signaling in proliferating chondrocytes leads to a shortening of the growth plate in part due to decreased cell proliferation, reduced TGFβ signaling results in an increased proliferation rate and an elongated growth plate. We also identify an interesting interaction between these two signaling pathways in a cell model of chondrogenesis.

## Results

### *Smad1^cKO/cKO^* mice show shortened growth plate

In the epiphyseal growth plate, *Smad1* is strongly expressed in proliferating chondrocytes [23]. *Smad1* null mice (*Smad1^−/−^*) die by 10.5 days post coitum (dpc) from defects in morphogenesis and proliferation of extra-embryonic tissue [24]. Therefore to analyze the Smad1 function in cartilage we used a conditional knockout strategy to specifically delete *Smad1* in the growth plate by crossing mice with a floxed Smad1 allele to transgenic mice carrying *Cre recombinase* driven by the type II collagen promoter (*Col2a1-cre*) [25]. To determine the efficiency of the *Smad1* deletion, DNA extracted from mouse tail or ribcage was used as template in a PCR with primers flanking the two loxP sites. A 350 bp product specific for the *Smad1* deletion confirmed the *in vivo* efficiency of Cre-mediated recombination in cartilage (**Figure S1B**).

To further verify that the conditional deletion was achieved, semiquantitative RT-PCR was performed using cDNA generated from P1 rib cartilage RNA (**Figure S1C**). *Smad1* expression was only observed in controls but not in the cartilage of *Smad1^cKO/cKO^* mice, indicating that *Smad1* is effectively deleted in proliferating chondrocytes. Due to the redundancy of *Smad1* and *Smad5* expression and function, these mice were further bred with *Smad5^+/−^* heterozygous mice to incrementally decrease overall BMP/Smad-signaling. Because of the embryonic lethality of Smad5^−/−^ mice, we did not further generate *Smad ^cKO/cKO^;Smad5^−/−^* mice so that we could study the postnatal phenotype in detail. Also it is now known that a conditional KO of *Smad1* and *Smad5* in chondrocytes leads to severe chondrodysplasia [13] and this approach enabled us to study subtle alterations in signaling during development and homeostasis. We were unable to detect any significant differences between WT, *Smad1^cKO/cKO^* and *Smad1^cKO/cKO^;Smad5^+/−^* mice by weight measurements (data not shown). Skeletal preparations with Alcian Blue and Alizarin Red staining showed no patterning defects in these mice (data not shown).

**Figure 1:**
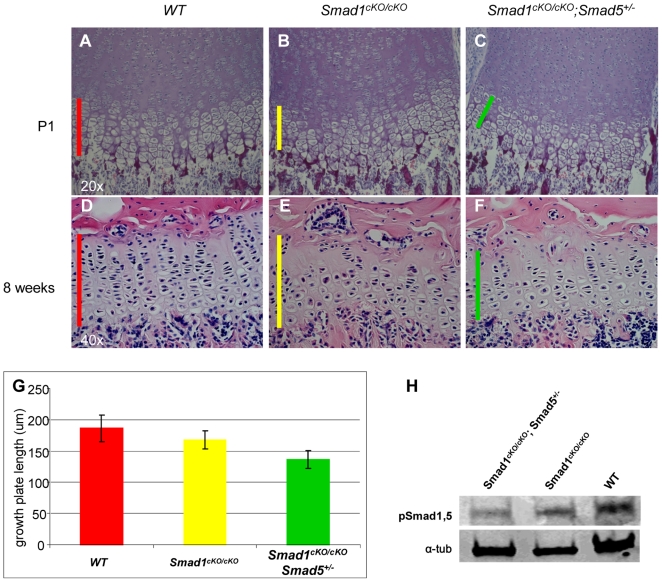
Shortened and disorganized growth plate in mice with disrupted BMP signaling. (A–C) Growth plate sections of long bones of P1 and (D–F) eight weeks old mice stained with Hematoxylin and Eosin. (G) Quantification of growth plate length (3 data points in each 20 sections of two mice per genotype, P = <0.001). cKO: conditional knock out; um: micrometer; WT: wild type. (H) Compared to WT control, *Smad1^cKO/cKO^* and *Smad1^cKO/cKO^;Smad5^+/−^* primary chondrocytes exhibit blunted response to rhBMP2 treatment, as revealed by decreased pSmad1,5 levels in the Western blots. α-tubulin is used as loading control.

Although the *Smad1^cKO/cKO^* and *Smad1^cKO/cKO^;Smad5^+/−^* mice show no gross growth phenotypes, to evaluate a subtle growth plate phenotype we next analyzed the long bone of these mice by a histological evaluation (**Figure 1A–C**). In P1 mice, our analysis demonstrated a significantly shortened growth plate in *Smad1^cKO/cKO^* mice (168.2+/−14.4 µm), and for *Smad1^cKO/cKO^*;*Smad5^+/−^* (136.8+/−14.5 µm) vs. WT littermates (186.3+/−21.5 µm) (**Figure 1G**). This result suggested a dosage requirement for BMP/Smad signaling during growth plate development. In eight weeks old adult mice, we observed similarly shortened growth plates in *Smad1^cKO/cKO^* and *Smad1^cKO/cKO^;Smad5^+/−^* mice supporting a continued postnatal requirement in postnatal growth plates (**Figure 1D–F**). To test whether the deletion of the Smad1 and Smad5 allele in cartilage can leads to a decreased response to BMP signaling, we detected pSmad1,5 level by Western blots in the rhBMP2 treated primary chondrocytes derived from *Smad1^cKO/cKO^* and *Smad1^cKO/cKO^;Smad5^+/−^* mice. As predicted, a distinctive decrease of pSmad1,5 level were detected in *Smad1^cKO/cKO^* chondrocytes, and the decreases is more pronounced in *Smad1^cKO/cKO^;Smad5^+/−^* mice, suggesting that lost of Smad1 and Smad5 allele effectively inhibit BMP canonic signaling (**Figure 1H**).

### *Smad1^cKO/cKO^;Smad5^+/−^* mice show no significant differentiation defect but decreased proliferation in chondrocytes

**Figure 2:**
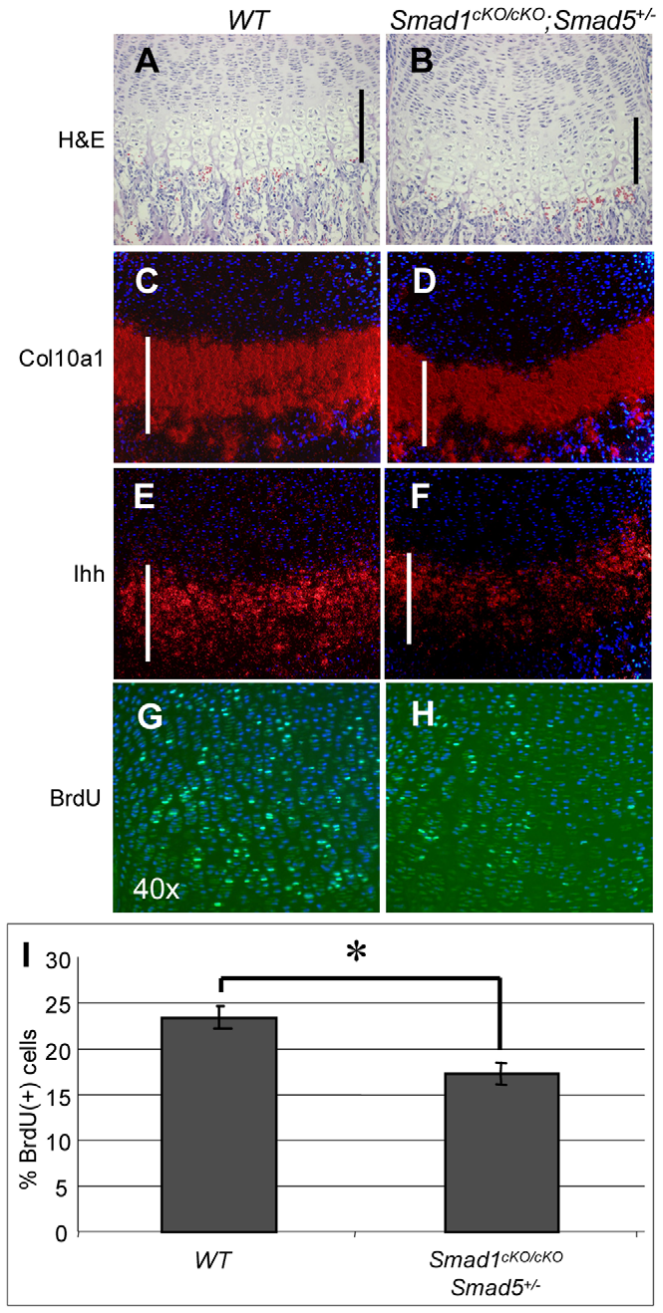
Decreased chondrocytes proliferation, shortening of pre-hypertrophic and hypertrophic zone in BMP signaling deficient mice. Shortened prehypertrophic and hypertrophic zone due to decreased proliferation in *Smad1^cKO/cKO^;Smad5^+/−^* mice compared to WT control. (A and B) Hematoxylin and Eosin staining, *In-situ* hybridization with (C and D) *Col10a1* and (E and F) *Ihh* probes showed that both the hypertrophic and prehypertrophic zone are shorter in *Smad1^cKO/cKO^;Smad5^+/−^* mice compared to WT control (indicated by white bars in each panel). (G–I) BrdU staining revealed a decreased proliferation rate in *Smad1^cKO/cKO^;Smad5^+/−^* compared to WT control (P<0.02).

To corroborate the reduced length of the prehypertrophic and hypertrophic zones, *in situ* hybridization for *Ihh* and *col10a1* was performed on hind limb sections, respectively. We found that both of the prehypertrophic and the hypertrophic zones in *Smad1^cKO/cKO^;Smad5^+/−^* mice were shortened and that *Ihh* expression was reduced in comparison to the wild type control mice (**Figure 2A–F**). By quantitative real time PCR, we observed a trend of decreased expression of *Ihh* and *Col10al* (data not shown). We further performed SafraninO staining as a qualitative assessment of extracellular matrix (ECM) but were unable to detect significant differences in ECM content in *Smad1^cKO/cKO^* or *Smad1^cKO/cKO^;Smad5^+/−^* mice (data not shown). These data suggest that a single allele of *Smad5* was sufficient to maintain the differentiation program of chondrocytes, while per previous report, complete loss of *Smad1* and *Smad* 5 in cartilage led to a severe chondrodysplasia with altered chondrocyte differentiation [13].

To understand whether the changes in chondrocyte proliferation contribute to the aforementioned growth plate phenotype in these animal models, we performed a BrdU incorporation assay to determine the proliferation status of *Smad1^cko/cko^* and *Smad1^cko/cko^;Smad5^+/−^* mice. In the femurs of P1 *Smad1^cKO/cKO^;Smad5^+/−^* mice, proliferation of chondrocytes was significantly reduced by 23.4% compared to wild type controls, while chondrocyte proliferation of *Smad1^cKO/cKO^* mice was also decreased by 17.3% compared WT mice. (**Figure 2** **G–I**). Hence the severity of the proliferative defect was inversely correlated with the number of *Smad1* and *Smad5* alleles remaining in cartilage.

### *Col2a1-ΔTβRI* transgenic mice exhibit an elongated growth plate

To study loss of function or impaired function of TGFβ signaling in the growth plate we generated transgenic mice that overexpress the dominant negative TGFβ receptor type I (ΔTβRI) specifically in proliferating chondrocytes by the Collagen 2a1 (Col2a1) promoter. The ΔTβRI is a truncated protein, retaining the ligand binding domain and the transmembrane domain, but lacking the activating serine/threonine kinase domain. Therefore, it can competitively bind to TGFβ ligands to act in a dominant negative fashion on wild type receptor-ligand interactions. In this manner, it acts as a decoy receptor by interacting with the ligand and type II receptors, and prevents the ligands' access to a functional receptor complex, thereby blocking TGFβ signaling (**Figure S2A**). Three founder mice were obtained (**Figure S2B**); the two founders with the highest expression were used to establish lines for phenotypic studies.

**Figure 3:**
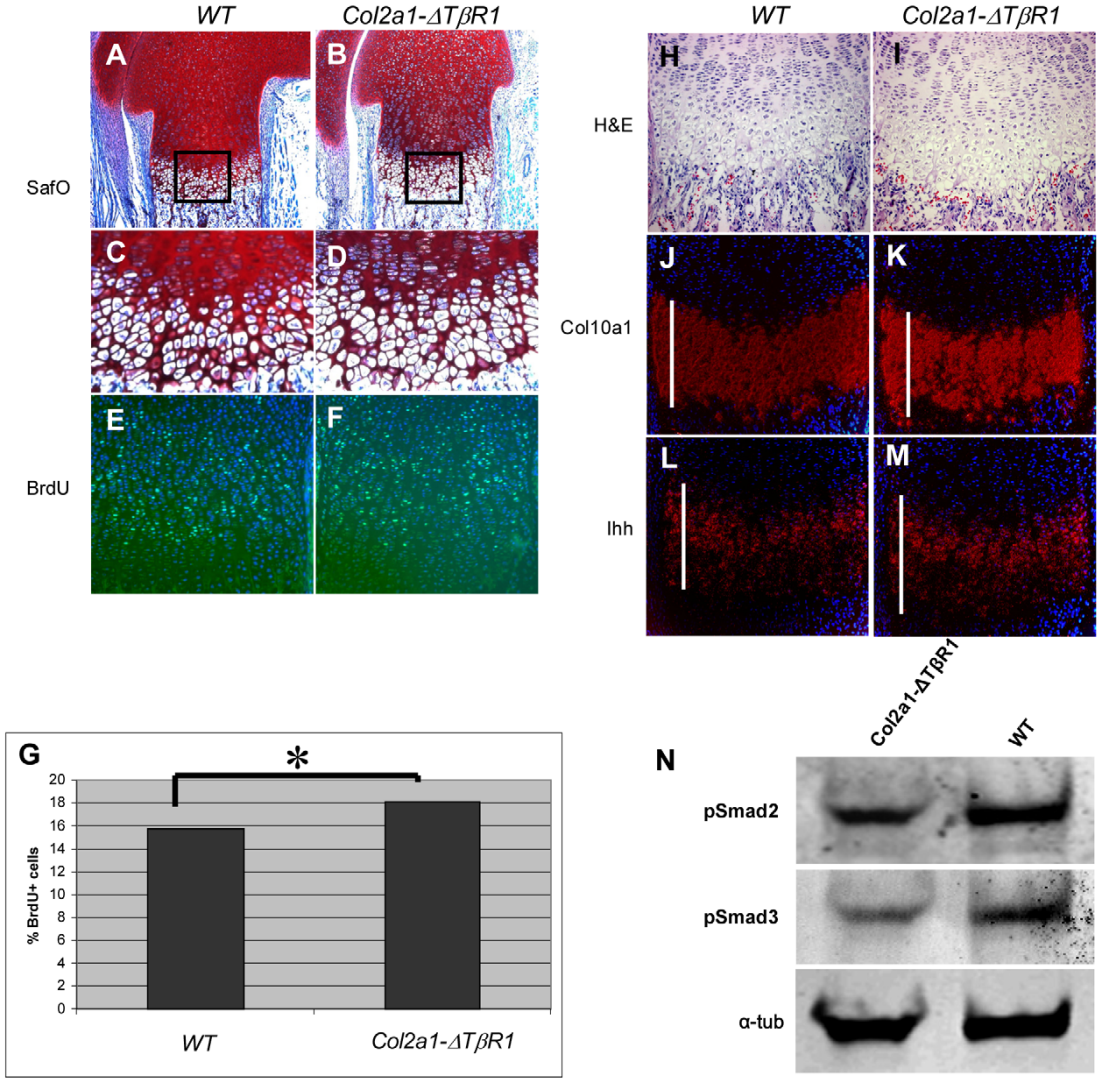
*Col2a1-ΔTβR1* transgenic mice exhibits elongated growth plate and increased proliferation of chondrocytes. Longer prehypertrophic zone, defective ECM production, and increased proliferation can be observed in *Col2a1-ΔTβR1* mice. Safranin O staining of (A) WT control and (B) the *Col2a1-ΔTβR1* mice (TG) shows a slight lengthening of the growth plate in the TG animals as well as a significantly impaired extracellular matrix production. (C–D) Also, a higher cell density in TG proliferating zone is detected. (E–G) TG proliferating zone exhibits increased BrdU positive cells compared to WT control (*P value = 0.02). While *in-situ* hybridization with *Col10a1* (J and K) does not show an increase in the size of the hypertrophic zone, *in-situ* hybridization with a probe for *Ihh* (L and M) demonstrates an elongation of the prehypertrophic zone (indicated by white bars in each panel). (H and I) Consecutive sections stained with Hematoxylin and Eosin. (N) Compared to WT control, *Col2a1-ΔTβR1* primary chondrocytes exhibit blunted response to to rhTGFβ1 treatment, revealed by decreased pSmad2 and pSmad3 levels in the Western blots. α-tubulin is used as loading control.

By Alcian blue and Alizarin red staining of the skeletons, a mild reduction in the size of the skeleton (data not shown) was noted, but there were no patterning defects observed in the P1 transgenic mice. Consistent with the smaller skeletal size, newborn transgenic mice demonstrated a statistically significant lower weight as compared with their wild type littermates (data not shown). Interestingly, in contrast to the loss of BMP signaling model, the growth plates of mice carrying the *Col2a1-ΔTβRI* construct were slightly elongated at P1 (**Figure 3 A–C**) and at 4 weeks (**Figure S2C and D**). To test whether the overexpression of *ΔTβRI* in cartilage can leads to a decreased response to TGFβ signaling, we detected pSmad2, and pSmad3 levels by Western blots in the rhTGFβ1 treated primary chondrocytes derived from *Col2a1-ΔTβRI* Tg mice. The result showed a distinctive decrease of pSmad2 and pSmad3 levels in the Tg chondrocytes, suggesting that *ΔTβRI* acts dominant-negatively and can effectively inhibit TGFβ canonic signaling (**Figure 1H**).

### C*ol2a1-ΔTβRI* transgenic mice show expanded prehypertrophic zone and increased chondrocytes proliferation

To further characterize the elongated growth plate phenotype in *Col2a1-ΔTβR1* transgenic mice, we evaluated markers of chondrocytes differentiation by *in situ* hybridization. We were unable to detect any significant changes using a hypertrophic zone-specific *Col10a1* probe (**Figure 3** **J and K**). However, the *Ihh* positive prehypertrophic zone was expanded in the transgenic mice (**Figure 3 L and M**). Moreover, the intensity of *Ihh* hybridization signal was qualitatively increased in the transgenic growth plate. We cannot exclude that the lack of phenotype in the hypertrophic zone might be due to lack of expression of the transgene in this region given that the Col2a1 promoter is inactive here.

We next evaluated the change of chondrocyte proliferation in the proliferating zone by BrdU incorporation assay. The *Col2a1-ΔTβRI* transgenic mice exhibit increased proliferation in chondrocytes compared to WT controls (p<0.005, **Figure 3E–F**). Here, we found that decreased TGFβ signaling *in vivo* led to increased chondrocyte proliferation, suggesting that TGFβ signaling is an inhibitor of proliferation of chondrocytes at least in this context.

Because TGFβ signaling is a key regulator of ECM synthesis and deposition, we next performed Safranin O staining on distal femurs in wild type controls, heterozygous, and homozygous *Col2a1-ΔTβRI* mice. We found that in the transgenic mice, ECM was remarkably decreased (**Figure 3 A–D**). At the same time, the cellular density of proliferating chondrocytes was also significantly increased consistent with the increase in chondrocyte proliferation.

### BMP2 can inhibit TGFβ signaling, while TGFβ1 enhances BMP signaling *in-vitro*

The interaction between BMP and TGFβ signaling has been known to be a critical determinant of various programs of organogenesis and homeostasis. The output of this crosstalk is complex and context dependent due to the fact that BMP and TGFβ can be antagonistic in some tissues, but synergistic in others [26], [27], [28]. Abundant published data support the importance of BMP and TGFβ as regulators in chondrogenesis and growth plate homeostasis [29], [30]. However, direct interaction of these two signaling pathways has not been clearly studied in cartilage. Hence, we attempted to further study this issue using an *in vitro* model of chondrogenesis, i.e., the ATCD5 rat chondrogenic cell line. Using BMP and TGFβ responsive luciferase reporter constructs (ld1- or SBE-Luciferase respectively) we evaluated the effects of combined BMP and TGFβ ligand on transfected, undifferentiated ATDC5 cells. After serum starvation and treatment 8 hours with human recombinant BMP and/or TGFβ (rhBMP, rhTGFβ respectively), cells were harvested for luciferase activity assay and Western blots.

**Figure 4:**
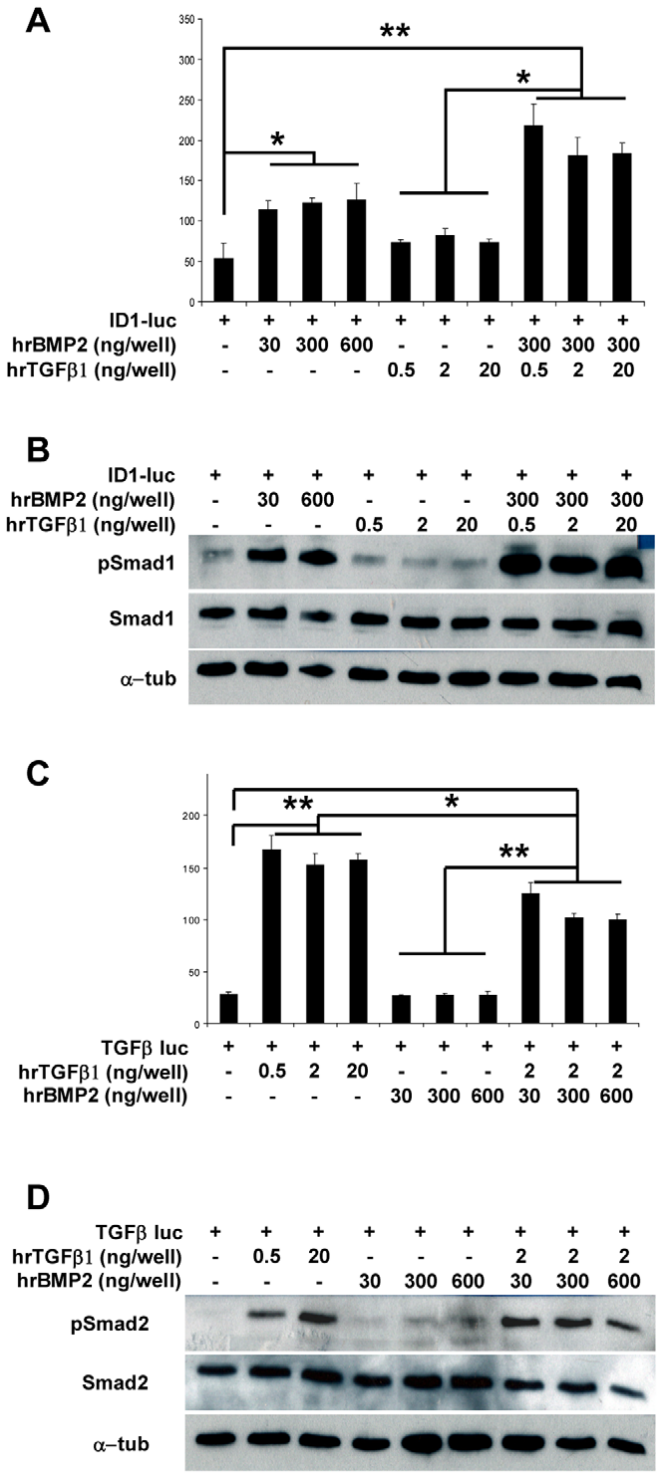
Mutual regulation between BMP and TGFβ signaling pathways in ATDC5 cells. In ATDC5 cells, TGFβ is able to enhance BMP signaling, while BMP2 has an antagonistic effect on TGFβ signaling. (A) The Id1-Luciferase reporter is significantly activated by different amounts of human recombinant (hr) BMP2 (30, 300 and 600 ng). The addition of hrTGFβ1 (0.5, 2 or 20 ng) leads to further increase in the luciferase activity. (B) Western Blot with BMP specific phospho-Smad1/5/8 confirmed the results. The amount of total Smad1of all samples under any treatment scheme is not significantly changed. (C) SBE-Luciferase reporter is significantly activated by different amount of rhTGFβ, while the addition of BMP2 decreases the overall TGFβ activity. (D) Western Blot using phospho-Smad2 antibody confirmed these results.

Both reporter systems responded respectively to BMP or TGFβ treatment and we found that the rhTGFβ1 treatment alone could not activate the BMP reporter. Similarly, treatment with recombinant BMP2 alone was not able to induce TGFβ reporter. But interestingly, TGFβ1 and BMP2 co-treatment led to an elevated BMP reporter activity when compared with the same amount of BMP2 alone. Moreover, this co-treatment was able to repress TGFβ signaling significantly in comparison to treatment with TGFβ alone (**Figure 4** **A and C**). These effects were dose responsive for both BMP2 (30, 300 or 600 ng/well) and TGFβ (2, 10 or 20 ng/well) concentration. These results were independently confirmed by western blots for p-Smad1/5/8 and p-Smad2 on the cell lysate, with total Smads1/5/8 and Smad2 as controls (**Figure 4** **B and D**).

## Discussion

The focus of this work has been compare the effects of BMP and TGFβ signaling and interactions in chondrocytes and in postnatal cartilage. The essential requirements for these respective pathways have been successfully generated by strong loss of function phenotypes. However, because of early lethality of complete loss of signaling models, it was difficult to assess their requirements postnatally during homeostasis. To circumvent this problem, we generated incremental deletions of *Smad1* and *Smad5* to achieve a partial deficiency of BMP signaling in chondrocytes. Our *in vivo* data showed that a single allele of Smad5 was sufficient to maintain postnatal growth and skeletal development suggesting that the wild type expression of *Smad1* or *Smad5* alone may not be the rate limiting step of BMP signal transduction. This also supports strong functional redundancy between Smad1 and Smad5 during chondrogenesis. However, when we looked closely at the growth plate phenotype, we observed a shortened and mildly disorganized growth plate in the *Smad1^cKO/cKO^* and *Smad1^cKO/cKO^;Smad5^+/−^* mice. This showed that by sequentially reducing total BMP-specific R-Smads, we were able to attenuate BMP signaling sufficiently to affect growth plate development and homeostasis while also elucidating an underlying cellular defect. Based on the recent published data by Retting et. al., complete deletion of *Smad1* and *Smad5* in chondrocytes leads to a dramatic lethal chondrodysplasia, suggesting a critical requirement of Smad1 and Smad5 during development [13]. In our studies, the mouse models of partial depletion of BMP signaling did not produce a strong chondrodysplasia enabling us the opportunity to study effects of loss of BMP function in adult animals and to compare loss of function of BMP versus TGFβ signaling at later developmental stages.

Our study showed that the decreased proliferation in the *Smad1^cKO/^* ^cKO^ or *Smad1^cKO/^* ^cKO^ *;Smad5^+/−^* proliferating chondrocytes contributes to the shortened postnatal growth plate. Canonical BMP signals can promote proliferation and differentiation in prehypertrophic chondrocytes, leading to the expression of *Ihh* in the prehypertrophic zone. Ihh then signals back via Ptc and Gli, leading to the expression of PTHrP, which in turn is able to keep chondrocytes in the proliferative pool [5], [31]. These data suggest that the disrupted BMP signaling in proliferating chondrocytes of the *Smad1^cKO/^* ^cKO^ or *Smad1^cKO^* ^/cKO^ *;Smad5^+/−^* mice inhibits proliferating chondrocytes differentiating to prehypertrophic chondrocytes. The observed proliferative phenotype may be related to down regulated PTHrP. One limitation of this approach is the lack of direct effect on potential Smads independent BMP signaling. But our and Retting et. al.'s findings suggest that MAPK pathway may not be essential for chondrocyte differentiation and maturation [13], since only one copy of Smad1 or 5 is enough to conserve an almost normal phenotype. The complete knock out of the canonical arm of the pathway leads to severe chondrodysplasia even though the non-canonical pathway is completely intact in this model.

The importance of TGFβ signaling during chondrogenesis and homeostasis has also been studied for decades but the conclusions of these studies have differed depending on the studies or disease models. This reflects the complex context-dependent regulation of TGFβ at both tissue and cellular levels. TβRI and TβRII are highly expressed in proliferating and maturing chondrocytes as well as the perichondrium in the growth plate [19]. To study the role of TGFβ signaling in the proliferating chondrocytes, we generated a transgenic mouse overexpressing a dominant-negative form of the TβRI to disrupt TGFβ signaling early in the differentiation program [32], [33]. Due to the high turn over rate of TGFβ receptors through rapid internalization and ubiquitin-dependent downregulation [34], we expect that the dominant- negative TβRI is predominantly expressed in proliferating chondorcytes and not in the hypertrophic chondrocytes. Our *in vivo* studies revealed that depletion of the TGFβ specific pathway leads to an overall longer growth plate due to an elongated proliferating zone and expanded *Ihh* positive prehypertrophic zone. We further detected significantly increased proliferation of chondrocytes and decreased extracellular matrix deposition in the proliferating zone of the transgenic mice. These data suggest that TGFβ signaling inhibits proliferation as well as the initiation of hypertrophy of chondrocytes.

Our finding is in line with some earlier studies. Mice that express a dominant negative TGFβ receptor type II under the control of a metallothionein promoter in skeletal tissues showed severe skeletal defects, including a disorganized growth plate with a prolonged prehypertrophic and hypertrophic zone [35]. The E-Selectin ligand-1 (ESL-1) knockout mice exhibit higher TGFβ bioavailability, and show shortened growth plate and growth retardation [36]. Moreover, *Ltbp-3* null mice, which show decreased TGFβ bioavailability in the skeleton, display both wider zone of prehypertrophic and hypertrophic zones in synchondrosis compared to wild type littermates [37]. Because the *Col2a1* promoter in our mice is not expressed in the hypertrophic chondrocytes, and the high tune over rate of TGFβ receptors, a notable phenotype in the hypertrophic zone is not detected.

The overlapping expression patterns of the BMP and TGFβ receptors, ligands and Smads suggest a potential interaction between BMP and TGFβ signaling in the developing and postnatal growth plate. To understand this possible interaction we used a chondrogenic cell line (ATDC5) and treated the cells with hrBMP2 and/or hrTGFβ1. The results showed that TGFβ1 significantly enhances BMP signaling, while in contrast, BMP2 inhibited TGFβ signaling. The negative regulatory role of BMP in TGFβ signaling has been demonstrated in several models, for example in the murine model systems for kidney disease [38] and in mouse pulmonary myofibroblast [39]. One explanation might be the competitive occupation of common downstream effectors such as Smad4. We detected a similar degree of Smad4 expression in treatment combinations (data not shown), but competitive inhibition would not necessarily change the total amount of Smad4. Another explanation could be that the induction of inhibitory Smad6 or Smad7 by BMP2 leads to the inhibition of the TGFβ signaling pathway, but again we could not detect any obvious differences in Smad6 and Smad7 protein levels (Data not shown). These data would suggest that the crosstalk between BMP and TGFβ signaling pathways in chondrogenic cells may depend on other mechanisms. Another possibility may also be the differential effects of canonical vs. noncanonical signaling in the specification of a cellular phenotype.

TGFβ acting as an activator of BMP signaling in chondrogenic cells is surprising to us, because it contradicted what has been established in osteogenic cell experiments. It has been reported that BMPs can promote expression of osteoblast differentiation markers in MC3T3 cells and lead to mineralization [40]. However, TGFβ1 cannot induce the expression of these osteogenic markers, but inhibits expression of osteocalcin and alkaline phosphatase stimulated by BMP2 treatment [40]. Another study showed that BMP3 and TGFβ1 have antagonistic effects on both proliferation and differentiation of human bone marrow stromal cells [41]. Moreover, BMP 2 was found to be able to induce osteogenic and chondrogenic phenotypes in adipocyte stem cells, which can be inhibited by the simultaneous TGFβ1 treatment [42].

However, in some other cell model systems, TGFβ was identified as acting synergistically with BMP signaling. For example, TGFβ can directly induce Smad1 phosphorylation in endothelial cells, by forming complexes between TβRII and ALK1, thus leading to stimulation of cell proliferation and migration [27]. Besides, TGFβ induced Smad1 phosphorylation was also identified in the C2C12 cells, keratinocytes, MEFs and HepG2 cells, suggesting that the synergetic effect of TGFβ on BMP signaling may exist in many other tissues during tissue development and homeostasis [27]. We expect that a similar signaling crosstalk may also be present in the chondrogenic cells. It is worthy to mention that in our experiment, TGFβ treatment alone could not induce Smad1 phosphorylation in the ATDC5 culture. This suggests that BMP may be required to potentiate a cellular context for the ATDC5, which allows TGFβ to stimulate Smad1 phosphorylation. For example, this potentiation may produce specific receptor complex or other signaling components that are required for TGFβ to stimulate Smad1 activation. This aspect is of considerable interest and deserves further studies.

Overall, this study provides new insight into the interaction of BMP and TGFβ signaling during growth plate homeostasis and endochondral ossification. Moreover, we found evidence for a crosstalk between these two signaling pathways, which differs from that as identified in bone. BMP2 is able to inhibit TGFβ1 signaling, while TGFβ1 can significantly increase BMP2 signaling in chondrocytes *in vitro*. Why the growth plate might be in need of such a signaling feedback loop is still puzzling. It is somewhat similar to the Ihh/PTHrP signaling loop, where Ihh promotes PTHrP expression while PTHrP suppresses Ihh expression. This Ihh/PTHrP feedback loop helps to define the balance between proliferating and hypertrophic cells and regulates the temporal progression of endochondral ossification in the growth plate. Whether the BMP/TGFβ feedback loop we identified in the chondrogenic cell line acts by a similar mechanism remains to be further studied. It may act in part to regulate proliferation in the context of such a negative feedback loop.

## Materials and Methods

### Ethics Statement

Handling and euthanization of mice for the following experiments was carried out in compliance with federal laws and regulations. The protocol was approved by the Institutional Animal Care and Use Committee (IACUC, Permit Number: AN1506) at Baylor College of Medicine.

### Generation of TG mouse line, breeding and genotyping

Smad1 conditional knock out mice were obtained from Dr. E. Robertson [24]. The Smad1 conditional allele was detected by PCR using a primer pair flanking the second loxP-site (F: 5-GTC ATC TCC TTC TCG TAC AG-3, R: 5-GGC CTT TAC TTT CTC GTG AC-3). The PCR was performed on tail-DNA of adult mice or DNA from skin of newborn mice. The Smad5 null mice were obtained by Dr. A. Zwijsen and were genotyped as described before [43]. The transgenic mice expressing *Cre recombinase* under the control of the *col2a1* promoter were obtained from Dr. R Behringer and the genotyping was performed as described before [25].

The dominant negative TβRI mice were generated by cloning of the truncated cDNA of the ALK5 under the control of the 6 kb *col2a1* promoter (*Col2a1-ΔTβR1*) [44]. A Tyrosinase cassette was used for visual genotyping [45], and a WPRE regulatory element at the 3′ end of the cDNA was included to enhance the expression of the transgenic protein [46]. The genotype was verified using primers specific for WPRE sequence (F:5-TCT CTT TAT GAG GAG TTG TGG CCC-3; R: 5-ACT GAC AAT TCC GTG GTG TTG TCG-3). Three different founders were generated.

### Histology, von Kossa staining, SafraninO staining, RNA in-situ hybridisation (ISH), and BrdU incorporation assay

For histological analysis, limbs from 8 week old mice were fixed in formalin for 48 h. Decalcification was performed for 14 days in 10% EDTA (pH 7.2) at room temperature. Limbs of P1 mice were fixed with 4% PFA overnight. All samples were embedded in paraffin and sectioned at 5 µm thickness. The RNA *in-situ* hybridization was performed as described previously [47]. For the BrdU (Bromodeoxyuridine, Invitrogen) incorporation assay, P1 mice were injected with BrdU (50 µg/g body weight, IP) and sacrificed after 2 h. BrdU-Alexa-488 antibody (Invitrogen) was used to detect the incorporated BrdU.

The length of the growth plate was measured using established histomorphometric markers. Specifically, each femur section was measured at three positions: two at the edges one in the center. The starting point was set at the boundary between the round chondrocytes and flat chondrocytes, and the end point was chosen at the onset of mineralization (as shown in **Figure S1D**)[48]. 20 sections per mouse and two mice each phenotype was measured blindly.

### Primary Chondrocyte culture and ligand treatments

Ribs cages are isolated from P3 mice, and incubate with Ponase (2 mg/ml in PBS) at 37°C for 30 min to remove the attached soft tissues. The bony tissue from the rib cage is subsequently removed by digestion in collagenase D (3 mg/ml in α-MEM) for 2 hours. The cleaned rib cartilages are next digest overnight in collagenase D (1.5 mg/ml) with 5% fetal bovine serum. The dissociated chondrocytes are washed by PBS and culture in 3.5 cm cell culture dishes at 1×10^6^/dishes. After 24 hours, when the cell reach 70% confluency, 100 ng/ml rhBMP2 or 2 ng/ml rhTGFβ1 are added to the culture. The chondrocytes are lysed in 1 x lamellae buffer and subjected to Western blots.

### DNA transfection and reporter assays

ATDC5 cells were cultured in a 1∶1 mixture of Dulbecco's modified eagles medium and Hams F12-Medium (DMEM/F-12) containing 5% fetal bovine serum (FBS), 10 ug/ml human Transferrin, and 3×10^−8^ M sodium selenite. Transfections were done using Fugene6 (Roche) as transfection reagent. 0.4 µg of BMP-luciferase reporter (Id1-luc) or TGFβ-luciferase reporter (SBE-Luc) was used each well. All wells were co-transfected with 0.1 µg of a TK-Renilla luciferase plasmid (Promega) as an internal transfection control. Six hours after transfection, the transfection medium was replaced with serum starved medium. The following day, 30–600 ng recombinant hBMP2 (R&D systems) or 2–20 ng recombinant hTGFβ1 (R&D systems) was added and incubated for 8 hrs. The luciferase assay was performed using the Dual Luciferase Reporter Assay System (Promega). Data represent the average ratios of Firefly-luciferase to TK-Renilla-luciferase activity.

### Western Blot

Protein from cells was isolated using lysis buffer containing 100 mM potassium phosphate with 0.1% Triton X. A standard western blot was performed using the following polyclonal antibodies: anti-pSmad1,5,8 (1∶1000, Cell signaling), anti-pSmad2 (1∶1000, Cell Signaling), anti-total Smad2 (1∶1000, Zymed) anti-total Smad1 (1∶1000, Invitrogen) and anti- α-tubulin monoclonal antibody (1∶5000, Sigma). The blots were subsequently incubated in Peroxidase-conjugated secondary antibodies (anti-rabbit IgG, 1∶5000, GE Healthcare UK Limited; or anti-mouse IgG, 1∶5000, Bio-Rad), and visualized by ECL reagents(Amersham Biosciences) and X-film (Kodak) autography.

### Statistical analysis

Data are expressed as mean values +/− standard deviation. Statistical significance was determined using Student's t test and data were considered statistically significant with a P value<0.05.

## Supporting Information

Figure S1 Smad1 is specifically and effectively deleted in Chondrocytes. (A) Schematic drawing of the *Smad1* gene locus and PCR genotyping strategy. (B) Genotyping PCR with primer pair P1/P2 (B-i), the product size on WT DNA is 140 bp, with incorporated loxP site the PCR product is 190 bp. On DNA of heterozygous animals we detected a third product of 220 bp. Sequencing showed that it is a heteroduplex of the two described products. Genotyping PCR for *Cre recombinase* locus (product size 500 bp, B-ii) and PCR on cartilage DNA with primer pair P3/P2 (product size 350 bp, B-iii) showing that the part between the loxP sites is effectively floxed out by Cre recombinase. (C) Semiquantitative RT-PCR on rib cartilage cDNA. cDNA from an animal negative for the *Cre recombinase* allele is marked as -. cDNA from an animal carrying the expressing *Cre recombinase* is marked as +. As expected, the expression of *Smad1* in animals carrying the *Cre recombinase* allele is very low compared to the wt control. *Smad1* is almost completely floxed out, the faint product could result from contamination with cells, which are not expressing *Cre recombinase*. (D) Quantification of growth plate length of *WT, Smad1^fl/^f,l* and *Smad1^fl/fl^;Smad5^+/−^*mice. Three measure points (marked with 1,2 and 3) in the growth plate were used to determine the length of the growth plate. Starting point was set at the boundary between the round chondrocytes and flat chondrocytes, and the end point was chosen at the onset of mineralization. bp: base pair; fl: floxed; P: Primer.
(TIF)

Figure S2 Transgenic overexpression of the dominant-negative TGFβ receptor type I (*ΔTβRI*) specifically in chondrocytes leads to elongated growth plate. (A) Transgenic construct used to generate transgenic mice. The Tyrosinase mini gene was used for coat color genotyping. A woodchuck hepatitis virus posttranscriptional regulatory element (WPRE) followed by the human growth hormone polyadenylation signal (hGHpA) was added to increase the transcription efficiency. Two HS4 chicken insulators were added for preventing the unspecific expression influenced by flanking genomic sequence. (B) Detection of TGFβ receptor type I over expressions levels in the rib cartilage of three established TG lines using quantitative real-time PCR. (C and D) The phenotype of elongated growth plate still remains in four weeks old transgenic mice by Hematoxylin and Eosin staining. *Col2a1*: Collagen type II; hGHpA: human growth hormone polyadenylation signal; HS4: 2x1.2-kb insulator element derived from the chicken beta-globin locus; TG: Transgenic; Tyr: Tyrosinase mini gene; WPRE: woodchuck hepatitis virus posttranscriptional regulatory element; WT: wild type.
(TIF)
