# Supplementary figures and images for: Interaction of TGFβ and BMP Signaling Pathways during Chondrogenesis
Source: PLoS One. 2011 Jan 28;6(1):e16421. doi: 10.1371/journal.pone.0016421 (PMC3030581; doi:10.1371/journal.pone.0016421)

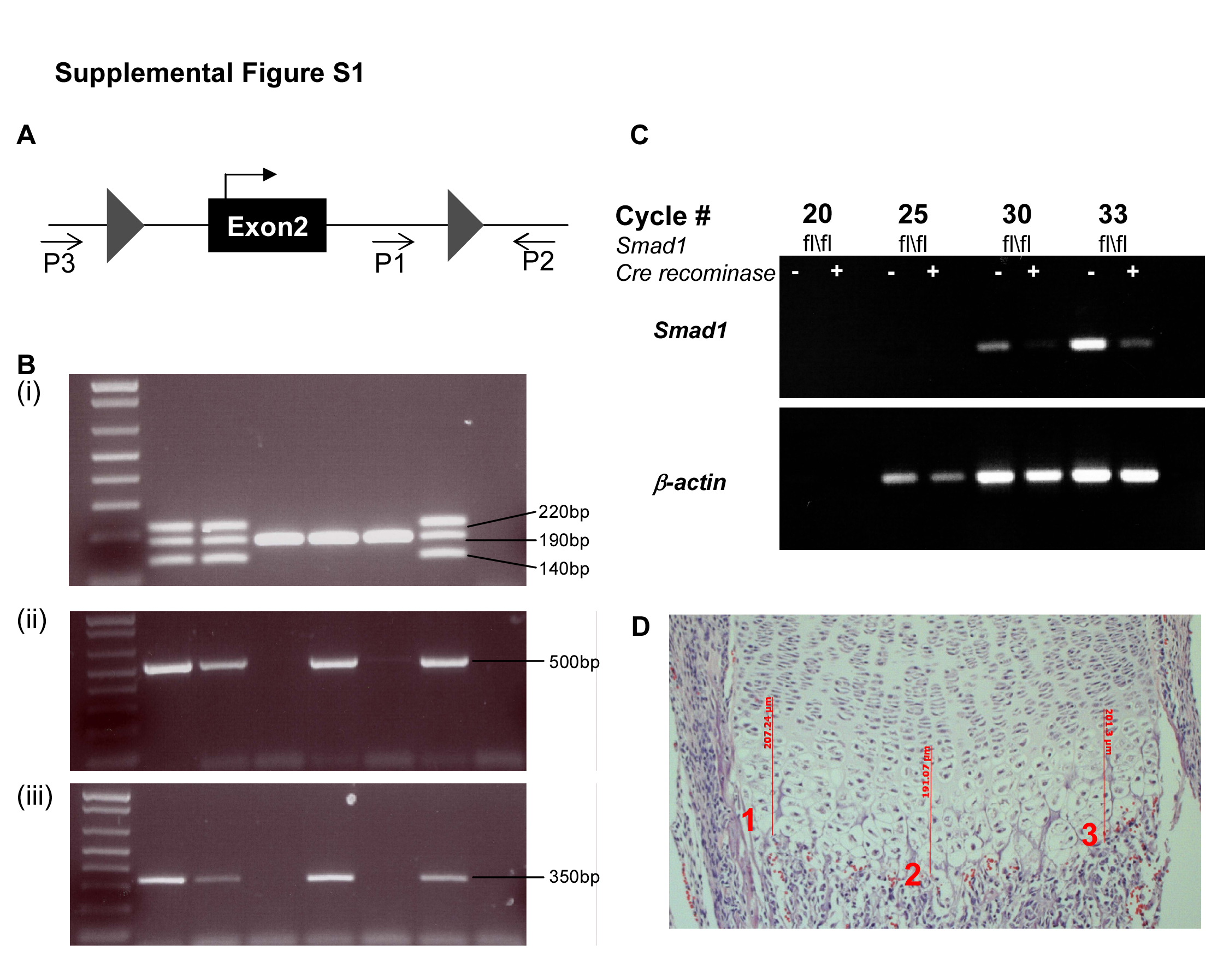

Supplement: Figure S1 — Smad1 is specifically and effectively deleted in Chondrocytes. (A) Schematic drawing of the Smad1 gene locus and PCR genotyping strategy. (B) Genotyping PCR with primer pair P1/P2 (B-i), the product size on WT DNA is 140 bp, with incorporated loxP site the PCR product is 190 bp. On DNA of heterozygous animals we detected a third product of 220 bp. Sequencing showed that it is a heteroduplex of the two described products. Genotyping PCR for Cre recombinase locus (product size 500 bp, B-ii) and PCR on cartilage DNA with primer pair P3/P2 (product size 350 bp, B-iii) showing that the part between the loxP sites is effectively floxed out by Cre recombinase. (C) Semiquantitative RT-PCR on rib cartilage cDNA. cDNA from an animal negative for the Cre recombinase allele is marked as -. cDNA from an animal carrying the expressing Cre recombinase is marked as +. As expected, the expression of Smad1 in animals carrying the Cre recombinase allele is very low compared to the wt control. Smad1 is almost completely floxed out, the faint product could result from contamination with cells, which are not expressing Cre recombinase. (D) Quantification of growth plate length of WT, Smad1fl/f,l and Smad1fl/fl;Smad5+/−mice. Three measure points (marked with 1,2 and 3) in the growth plate were used to determine the length of the growth plate. Starting point was set at the boundary between the round chondrocytes and flat chondrocytes, and the end point was chosen at the onset of mineralization. bp: base pair; fl: floxed; P: Primer. (TIF) [file pone.0016421.s001.tif]

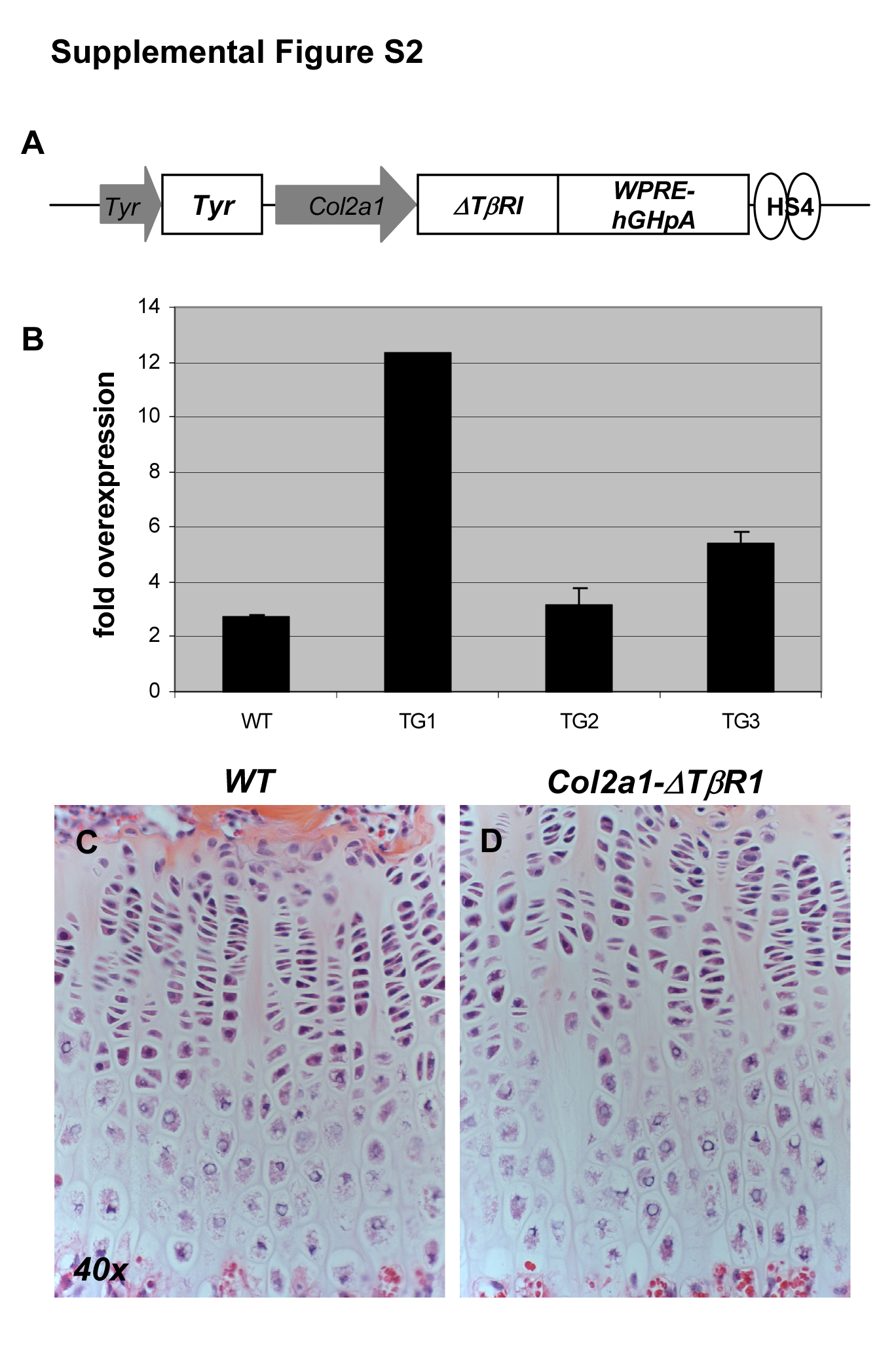

Supplement: Figure S2 — Transgenic overexpression of the dominant-negative TGFβ receptor type I (ΔTβRI) specifically in chondrocytes leads to elongated growth plate. (A) Transgenic construct used to generate transgenic mice. The Tyrosinase mini gene was used for coat color genotyping. A woodchuck hepatitis virus posttranscriptional regulatory element (WPRE) followed by the human growth hormone polyadenylation signal (hGHpA) was added to increase the transcription efficiency. Two HS4 chicken insulators were added for preventing the unspecific expression influenced by flanking genomic sequence. (B) Detection of TGFβ receptor type I over expressions levels in the rib cartilage of three established TG lines using quantitative real-time PCR. (C and D) The phenotype of elongated growth plate still remains in four weeks old transgenic mice by Hematoxylin and Eosin staining. Col2a1: Collagen type II; hGHpA: human growth hormone polyadenylation signal; HS4: 2x1.2-kb insulator element derived from the chicken beta-globin locus; TG: Transgenic; Tyr: Tyrosinase mini gene; WPRE: woodchuck hepatitis virus posttranscriptional regulatory element; WT: wild type. (TIF) [file pone.0016421.s002.tif]
